# Effects of transcutaneous electrical acupoint stimulation (TEAS) on postoperative pain in patients undergoing gastric and esophageal ESD surgery: a study protocol for a prospective randomized controlled trial

**DOI:** 10.1186/s12906-023-04075-9

**Published:** 2023-07-20

**Authors:** Xin-Lu Chang, Xu-Ming Liu, Li-Xin An, Jian-Yong Zheng, Ke Zhang

**Affiliations:** 1 Department of Anesthesiology, Beijing Friendship Hospital, Capital Medical University, No. 95 Yongan Road, Xicheng District, Beijing, 100050 China; 2 Department of Anesthesiology, Perking University Cancer Hospital, Beijing, China; 3 Department of Anesthesiology, Anning First People’s Hospital Affiliated to Kunming University of Science and Technology, Anning, China

**Keywords:** Endoscopic submucosal dissection (ESD), TEAS, Postoperative pain

## Abstract

**Background:**

Post-operative pain of endoscopic submucosal dissection (ESD) is always be overlooked and undertreated by endoscopists. However, the incidence of moderate to severe pain after ESD is as high as 44.9% to 62.8%, which can greatly affect the patient’s recovery, reduce their satisfaction, and extend their hospital stay. Transcutaneous electrical acupoint stimulation (TEAS) have been shown to reduce postoperative pain and enhance gastrointestinal (GI) function recovery in patients undergoing abdomen surgery. However, there is no evidence regarding on the effect of TEAS on post-operative pain and complications in patients undergoing ESD. Therefore, we aim to investigate whether perioperative TEAS treatment is superior to the sham acupuncture in terms of post-ESD pain and GI function recovery.

**Methods:**

This study is a prospective, randomized controlled trail, which is single-blinded and in single center. A total of 120 patients undergoing elective gastric and esophageal ESD surgery in Beijing Friendship Hospital, Capital Medical University, will be involved in this study. These individuals will be stratified according to the type of ESD surgery (i.e. gastric or esophageal procedure) and be randomly divided into two groups. L14, PC6, ST36 and ST37 will be stimulated at the TEAS treatment group, and the control group will receive simulation at four sham acupoints. The primary outcome is post-EDS VAS score at the time of entering PACU, 10 min, 20 min, 30 min, 1 h, 2 h, 4 h, 6 h, 18 h, 24 h, 48 h after the surgery. The secondary outcomes include the anesthesia-associated parameters, sedation score, nausea and vomiting score, shivering score, recovery of gastrointestinal function, satisfaction of patients to anesthesia, incidence of postoperative complications, QLQ-C30 life quality scale, and the economic indicators.

**Discussion:**

The results of this study will confirm that continuous preventive application of TEAS can alleviate the postoperative pain among patients with gastric and esophageal ESD surgery and accelerate the recovery of post-ESD gastrointestinal function.

**Trial registration:**

Chinese Clinical Trial Registry, ID: ChiCTR2100052837, registered on November 6, 2021. http://www.chictr.org.cn/showproj.aspx?proj=135892.

**Supplementary Information:**

The online version contains supplementary material available at 10.1186/s12906-023-04075-9.

## Background

Endoscopic submucosal dissection (ESD) is a minimally invasive treatment for early gastrointestinal neoplasms, with the advantages of histological en bloc mucosal resection, accurate pathologic diagnosis, low recurrence rate, and fast recovery after surgery [1–3]. However, we should not ignore the complications after ESD. In addition to the high incidence of intraoperative bleeding and perforation, complications such as postoperative abdominal distension, abdominal pain, nausea and vomiting are also common [2, 3]. Some studies showed that the moderate to severe pain after EDS surgery can appear at the incidence of as high as 44.9–62.8%, especially in the early postoperative period (within 1-4 h after surgery) [4]. The post-ESD pain will greatly affect the patient postoperative recovery, reduce patient satisfaction, and increase length of hospital stay and medical costs [2–5]. Unfortunately, the postoperative pain after ESD is always overlooked or underestimated, partly due to the excessive engagement of earlier day of discharge and enforced early mobilization in the ERAS protocols [5, 6].

Presently, there has been no unified conclusion on how to perform effective postoperative analgesia to patients with gastric and esophageal ESD surgery. Because such patients need to be discharged quickly, overdose of opioids are often not recommended. Some studies found that intravenous dexamethasone or lidocaine, or local infusion of bupivacaine and triamcinolone during ESD procedure can help to relieve post-ESD abdominal pain, while reducing the consumption of intraoperative opioids and decreasing patient body movement [7–9]. However, it requires to be studied on how long the adjuvant analgesic effect of the above perioperative drugs can last [10]. In conclusion, the ESD patients urgently need effective postoperative analgesia methods with little impact on patient functions.

As an important part of traditional Chinese medicine, acupuncture and moxibustion are widely used to regulate organ functions during perioperative period, including the treatment of postoperative pain [11]. Transcutaneous Electrical Acupoint Stimulation (TEAS) is an improvement on traditional acupuncture. It sends electrical pulses to acupoints through electrodes on the skin surface, which is non-invasive, easily operatable, and effective for pain relief [12]. In recent years, numerous studies have demonstrated the effect of TEAS on alleviation of postoperative pain, postoperative nausea and vomiting (PONV), enhancement of gastrointestinal function recovery, and reduction of stress reaction [11–13].

In our previous study, it was found that preventive continuous application of TEAS can reduce postoperative pain and promote gastrointestinal function recovery in patients undergoing laparoscopic resection of gastroesophageal tumors [14–16]. However, if TEAS have the same effect on postoperative pain of minimally invasive surgery such as ESD is remained unexplored. Therefore, this study tries to demonstrate that preventive applying TEAS to superior acupoints can reduce postoperative pain and promote the recovery of gastrointestinal function in patients undergoing gastroesophageal ESD surgery.

## Methods and design

This trial has been approved by the Ethics Committee of Beijing Friendship Hospital, Capital Medical University (Approval No: 2021-P2-315-01), and has been registered in the Clinical Trial Registry (Chictr) with number: ChiCTR2100052837 (see Additional file 1). The study will be implemented at Beijing Friendship Hospital and will be carried out in accordance with the principles of the Declaration of Helsinki (version Edinburgh 2000). Additional file 2 shows the SPIRIT 2013 Checklist, which this protocol is based on.

### Aim

This trail aims to confirm that the preventive application of TEAS in patients undergoing gastroesophageal ESD surgery can release postoperative pain while promoting recovery of gastrointestinal function. Moreover, we can provide clinical evidence for the inclusion of perioperative TEAS into enhanced recovery after surgery (ERAS) of gastrointestinal surgery.

### Trial design

**Fig. 1:**
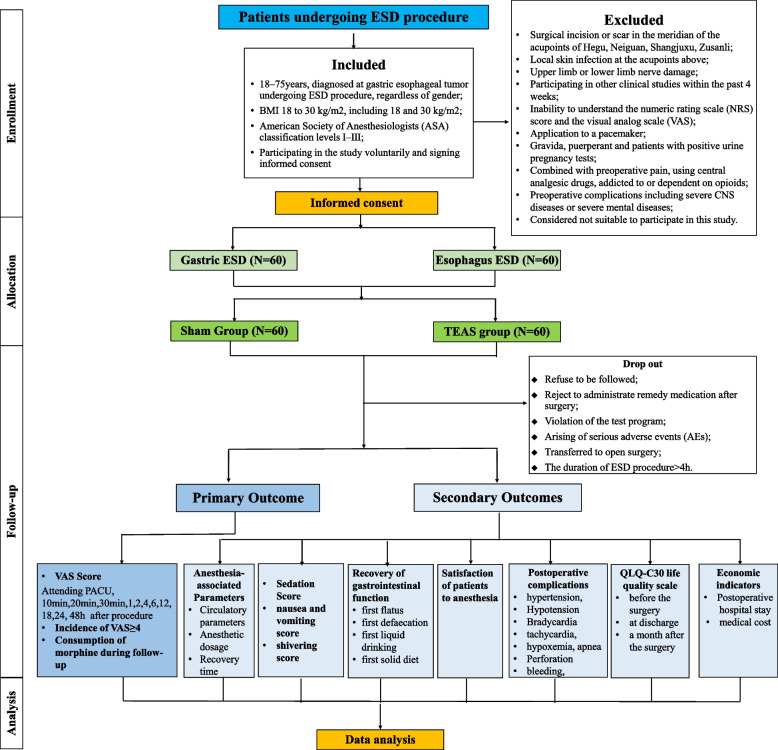
Consolidated Standards of Reporting Trials (CONSORT) diagram for this trail

This study is a single-center, prospective, single-blind, randomized, and controlled trial. A total of 120 patients undergoing elective gastric and esophageal ESD surgery in Beijing Friendship Hospital, Capital Medical University, will be stratified by the type of ESD surgery (i.e. gastric or esophageal procedure) at 1:1 ratio and be randomized into two groups: the sham group (Group S, *n* = 60) and the TEAS treatment group (Group T, *n* = 60). Each group has 30 gastric cases and 30 esophageal cases (Fig. 1). The observers will conduct screening according to the pre-standard plan. Data gathering will start from the collection of basic data and last until the end of follow-up (Table 1). According to the hospital regulations, compensation and post-trial care will be provided to those injured due to their participation in the trial.

**Table 1:**
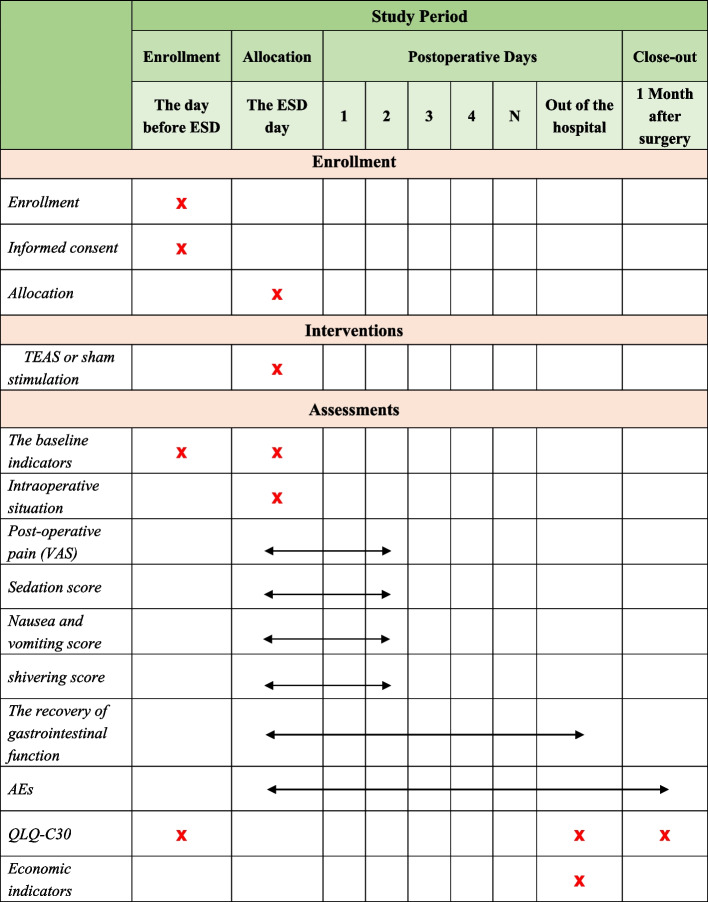
Standard Protocol Items: Recommendations for Interventional Trials (SPIRIT) Schedule for enrollment, interventions, and assessments

### Randomization and blinding

This is a single-blinded study. To minimize evaluation bias, the evaluators responsible for screening and outcome evaluation will be blinded to the grouping information. The anesthesiologist, and the acupuncturist who is in charge of the TEAS stimulation from the traditional Chinese department, will acknowledge each patient’s affiliation, but will be isolated to the research results. Patients will not be informed about their group assignment. Randomization will be performed by a computer-generated blocked randomization order. A nurse will generate the allocation sequence and prepare the sealed, numbered envelops. Each envelop will be unfolded by the anesthesiologist until the patient entering the operating room. Subjects will be randomly assigned to the sham group or the TEAS group with a ratio of 1:1 by the anesthesiologist according to these envelops. The case report forms (CRF) will be used to record all the original data needed.

### Study participants and recruitment

We will recruit 120 patients who are scheduled to undergo gastric or esophageal ESD procedure. These participants will be recruited from Beijing Friendship Hospital affiliated to Capital Medical University after they are up to the eligibility criteria and sign the informed consent. We enrolled the first patient on December 1st, 2021, and will end up recruiting on December 30th, 2023. Full communication and some small gifts such as keychains will be provided for patients to encourage the enrollment. All subjects will sign the informed consent form to participate in this clinical trial, allowing the researchers to collect and analyze their data.

***Inclusion criteria include the following:***

1. Aged 18–75 years, diagnosed at gastric or esophageal tumor undergoing ESD procedure, regardless of gender.
2. 18 kg/m^2 ^≤ BMI ≤ 30 kg/m^2^ [body mass index = weight (kg)/height (m)^2^].
3. American Society of Anesthesiologists (ASA) classification levels I–III.
4. Participating in the study voluntarily and signing informed consent.

***Exclusion criteria include the following:***

1. Surgical incision or scar in the meridian of the acupoints of Hegu (L14), Neiguan (PC6), Shangjuxu (ST37), Zusanli (ST36).
2. Local skin infection at the acupoints above.
3. Upper limb or lower limb nerve damage.
4. Participating in other clinical studies within the past 4 weeks.
5. Inability to understand the numeric rating scale (NRS) score and the visual analog scale (VAS) score.
6. Application to a pacemaker.
7. Gravida, puerperant and patients with positive urine pregnancy tests.
8. Combined with preoperative pain, using central analgesic drugs, addicted to or dependent on opioids.
9. Preoperative complications including severe CNS diseases or severe mental diseases.
10. Considered not suitable for this study.

***Discharge criteria are as follows:***

1. Requirement for participants to withdraw during the trail.
2. Rejection of administration of remedy medication after surgery.
3. Violation of the test program.
4. Arising of serious adverse events (AEs).
5. Transferred to open surgery.
6. The duration of ESD procedure > 4 h.

### Standard procedures

**Fig. 2:**
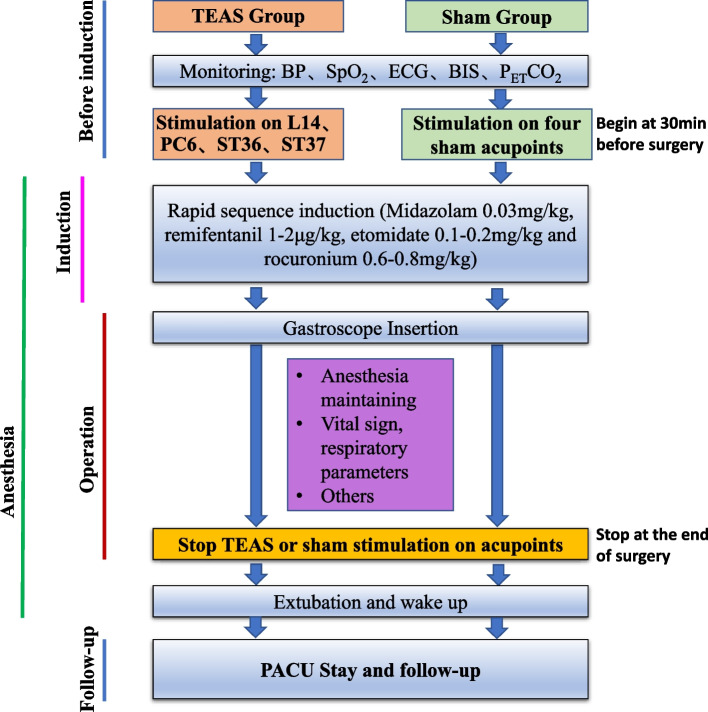
Perioperative protocol of anesthesia

The following approaches are suggested (Fig. 2):

1. The anesthesiologist will perform preoperative education on patients who meet the inclusion criteria and make sure they can understand and utilize VAS and NRS to evaluate their degree of pain and nausea when patients are admitted into operation room. Explaining to patients of the research purpose, process, risks and benefits, and other related contents. Guaranteeing that they are willing to adhere to trail schedule. Finally, ensuring that self-reported information sheet which is used in postoperative follow-up is issued and the informed consent is signed.
2. Electrocardiogram, blood pressure, pulse oxygen saturation and BIS will be performed.
3. The TEAS or sham stimulation will be conducted by the experienced anesthesiologist and acupuncturist at 30 min before the surgery until the end of the operation.
4. Rapid sequence induction is applied as anesthesia induction strategy. Midazolam 0.03 mg/kg, remifentanil 1-2 μg/kg, etomidate 0.1–0.2 mg/kg and rocuronium 0.6–0.8 mg/kg is used before intubation. When the patients lose their consciousness, mask ventilation is given. Mechanical ventilation will be performed after endotracheal intubation (tube size 7.5 for male and 7.0 for female).
5. Anesthesia is maintained with total intravenous infusion of propofol (4-6 mg/kg/h), remifentanil (0.05–0.2 µg/kg/min) and rocuronium (one third of the induced dose every 40 min). Anesthetic depth is controlled by the adjustment of infusion speed of intravenous propofol to maintain a bispectral index at 45–55 during procedure. The analgesia nociception index (ANI) is maintained at 50–70 by regulating the speed of remifentanil intravenous infusion. Respiratory parameters are adjusted to maintain PetCO_2_ at 35–45 mmHg. The infusion of sedative and analgesic agents are stopped after suture of the operative incision.
6. Under the premise of maintaining appropriate anesthesia depth, atropine 0.25–0.5 mg at one time is given if heart rate (HR) is under 50 times per minute and esmolol 0.2–0.5 mg/kg is administrated if HR is above 100 times per minute. If the mean blood pressure is below 65 mmHg or decrease by more than 20% of baseline, ephedrine 6 mg will be given. Tramadol 50 mg is applied for analgesia 30 min before the end of ESD surgery.
7. At the end of surgery, patients will be extubated and transferred to the postoperative care unit (PACU) in routine.
8. The Visual Analogue Scale (VAS) will be utilized to evaluate the extent of the pain of patients, and if VAS score ≥ 4 points or the patients asked for analgesia during the follow-up period, morphine 1 mg will be administered intravenously. The Numerical Rating Scale (NRS) is used to evaluate the degree of nausea. If NRS ≥ 7 or vomiting occurs, serotonin 3 receptor antagonist will be given. Concomitant medication administration during the trial period from the beginning of surgery to 2 days after the end will be recorded.

### Intervention

**Fig. 3:**
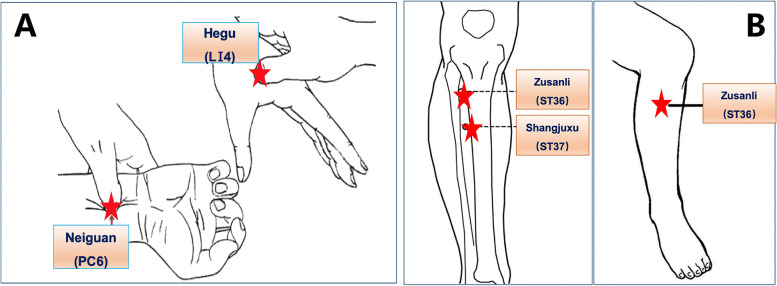
The location of acupoint. **A** The location of L14, PC6. *L14 is Hegu acupoint; PC6 is Neiguan acupoint*. **B** The location of ST36, ST37. *ST36 is Zusanli acupoint; ST37 is Shangjvxu acupoint*. This figure was drawn by the corresponding author of this manuscript Dr. Li-Xin An [14]. Permission was obtained

Patients in TEAS group will be treated bilaterally at four acupoints: Hegu (L14), Neiguan (PC6), Zusanli (ST36) and Shangjuxu (ST37). L14 is the primary acupoint of the hand yangming large intestine meridian, which is located on the dorsum of both hands between the first and second metacarpal bones (Fig. 3A). PC6 belongs to the pericardium meridian, located on the palmar side of the forearm, 2 cun above the rasceta, between the palmaris longus tendon palmar tendon and the flexor carpi radialis muscle tendon (Fig. 3A). ST36 and ST37 are acupoints of the Yangming stomach meridian. ST36 is situated at the lateral side of the lower leg, on the line of Dubi and Jiexi, 3 cun below Dubi and one finger width lateral to the anterior border of the tibia (Fig. 3B). ST37 is located 3 cun below ST36. The “cun” in traditional Chinese medicine generally refers to “the same body size”. The distance between the radial ends of the first and second transverse patterns of the patient’s middle finger is “1 inch”. Four fingers, excluding the thumb, are placed side by side and generally refer to “3 cun”. Self-adhesive electrodes with wires will be attached to the locations of these acupoints and will be connected to the HANS acupoint nerve stimulator (HANS-200A, Nanjing Jisheng Medical Technology Co., Ltd., China). The current frequency will be set at 2 and 100 Hz. A dense-dispersed wave will be applied, alternating the frequencies every 3 s. The stimulation intensity will be set at the level of maximum tolerance for patients. The TEAS stimulation will start at 30 min before the surgery until the end of the operation.

**Fig. 4:**
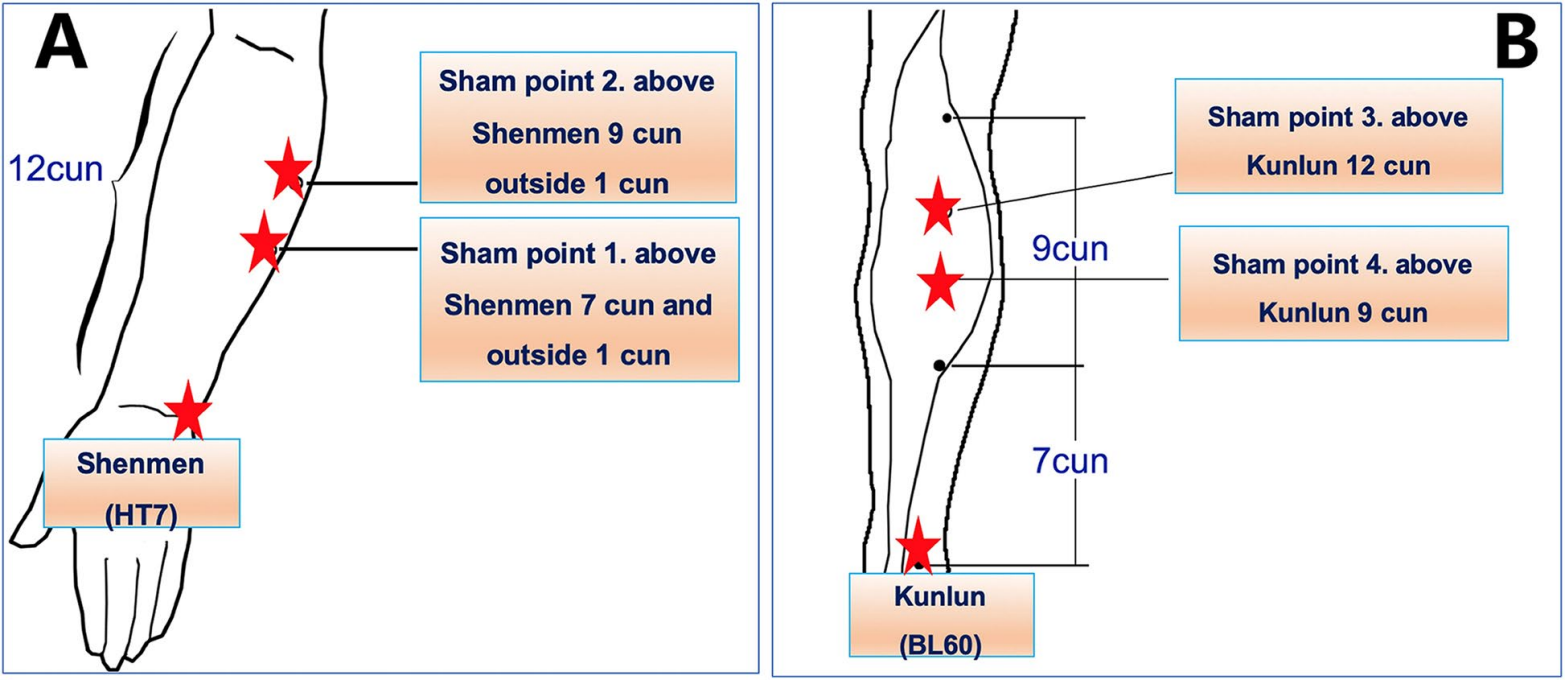
The location of sham point. **A** The location of sham point 1 and 2. *HT7 is Shenmen acupoint*. **B** The location of sham point 3 and 4. *BL60 is Kunlun acupoint*. This figure was drawn by the corresponding author of this manuscript Dr. Li-Xin An [14]. Permission was obtained

**Fig. 5:**
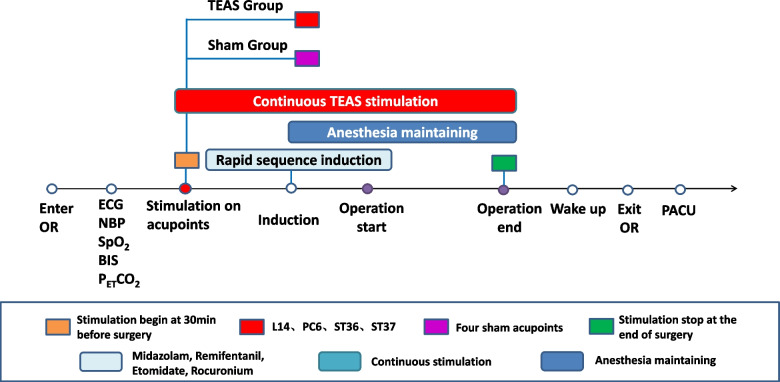
The administration of TEAS treatment protocol

In the control group, two of the sham points are at 7 cun above and 1 cun outside Shenmen (HT7), and 9 cun above and 1 cun outside HT7 (Fig. 4A). The other two sham points are located at 9 cun and 12 cun above the Kunlun (BL60) (Fig. 4B). Self-adhesive electrodes with wires will be also sticked to the sites of these acupoints and will be connected to the HANS acupoint nerve stimulator. However, no power will be supplied to the stimulator. Analog pulses will be used at the same time 30 min before the surgery until the end of the operation (Fig. 5).

### Outcomes

#### Primary outcome

The primary outcome is post-operative pain (VAS) score at the time of entering PACU, 10 min, 20 min, 30 min, 1 h, 2 h, 4 h, 6 h, 18 h, 24 h, 48 h after the surgery. The incidence of post-ESD VAS ≥ 4 and the consumption of morphine during follow-up will also be recorded.

#### Secondary outcomes

The secondary outcomes are as follows:

- ♦ ***Anesthesia-associated parameters:*** The systolic pressure, diastolic pressure and heart rate will be recorded before anesthesia induction, intubation, 30 min, 60 min after the start of surgery, at the end of the surgery and 5 min before and after extubation. The amount of intraoperative sedative and analgesia drugs consumption will be recorded. The time from the end of propofol infusion to the recovery of autonomous respiration, to extubation and to the eye open of patients will be recorded. The occurrence of dysphoria, nausea and vomiting will be also written down.
- ♦ ***Sedation score:*** Sedation score after complete awakening will be assessed at the moment of entering PACU, 10 min, 20 min, 30 min, 1 h, 2 h, 4 h, 6 h, 18 h, 24 h, 48 h after surgery.
- ♦ ***Nausea and vomiting score, shivering score:*** These score will be recorded at the moment of entering PACU, 10 min, 20 min, 30 min, 1 h, 2 h, 4 h, 6 h, 18 h, 24 h, 48 h after surgery.
- ♦ ***The recovery of gastrointestinal function:*** Time to first flatus, time to first defecation, time to first liquid drinking and tolerance of a solid diet will be recorded through self-assessment paper.
- ♦ ***Satisfaction of patients:*** Satisfaction of patients to anesthesia will be asked at 48 h before surgery.
- ♦ ***Incidence of postoperative complications:*** Postoperative hypertension, hypotension, bradycardia, tachycardia, hypoxemia, apnea, perforation, bleeding, aspiration pneumonia will be paid attention to and recorded.
- ♦ ***QLQ-C30 life quality scale:*** Patients will be requested to complete this scale before the surgery, at discharge and a month after the surgery.
- ♦ ***Economic indicators:*** Postoperative hospital stay and medical costs will be recorded after patients’ discharge.

### Data collection methods and monitoring

The observer, under the instruction of statistical professionals, gathers data including the basic information of patients enrolled, primary and second outcomes, incidence of complications, AEs etc. All the original data needed will be recorded accurately and completely in the case report forms (CRF). Any defective cases will be recorded, for example, in which patients forget their time to first flatus, first defecation, first liquid drinking and tolerance of a solid diet. All the period of this study will be monitored by Data Monitoring Committee (DMC), which consists of a doctor responsible for data collection and sorting, a statistician and a scientific research manager. DMC will be independent from the sponsor and competing interests. They will perform unblinding and weigh effectiveness and safety in the middle stage of this clinical study, and make the decision of continuing the trail, adjusting the protocol, or terminating the test, with communication to IRBs. At the end of the research, the distribution information, the original data and results will be presented to the scientific research management committee, who have access to the final trial dataset; they will be kept unknown to the public before the results are published.

### Sample size calculation

The primary outcome of this study is the postoperative VAS score. In accordance with the pre-experimental results, there will be no severe postoperative pain until 1 h after the surgery. Therefore, sample size was calculated based on the VAS score at 1 h after the ESD procedure. The mean VAS at 1 h in TEAS group and the sham group in the pre-experiment was 0.86 ± 1.14 and 1.68 ± 1.46, respectively. The PASS 11.0 software was used, with unilateral α = 0.05, β = 0.1 and power = 0.90. A total of 55 effective samples were estimated for each group. Considering of 10% patient loss, we set 60 cases for each group in this study. Eventually, we will involve 120 cases, including 60 patients in the TEAS group and 60 patients in the control group (30 in esophageal ESD and 30 in gastric ESD procedure). Our digestive endoscopy center performs 300–400 upper gastrointestinal ESD surgeries, so this trail period is set at 16 months consequently.

### Statistical analysis

Statisticians will negotiate with the research team to establish statistical analysis plans, develop databases, and perform statistics with the SPSS statistical analysis system. Statistics analyses include demographic and other baseline characteristics, postoperative pain degree analysis, statistical analysis of recovery time of gastrointestinal function, the incidence of complications and combined treatments, and comprehensive efficacy evaluation. Statisticians will use mean ± standard deviation (SD) to express measurement data that meet the normality test and use t-test to analyze. Non-parametric measurement data will be described as median and interquartile range (IQR), and Wilcoxon signed rank test will be applied. Enumeration data will be expressed as numbers and percentages. Pearson’s chi-squared test and Fisher’s exact test will be the methods applied for these data. Univariate logistic regression analysis will be applied to analyze risk factors which are reported to have possible correlation with VAS ≥ 4 in previous studies. Variables which are considered statistically significant (*P* < 0.05) will be selected for multivariable logistic regression to explore the association of those with post-ESD VAS ≥ 4. The Hosmer-Lemeshow test will be used to evaluate goodness-of-fit of the model. All *P* values above are unilateral and *P* < 0.05 will be considered significant. Subgroup analysis will be conducted in accordance with different procedure types. Intention to treat (ITT) principle will be applied to analyze all data. Because all the data will be collected during hospital stay by a conscientious observer (except for the last entry of QLQ-C30 life quality scale, which will be completed at a month after surgery), missing data can be mostly avoided. The mean imputation method will be used if missing data still preserves. The Capital Medical University statistical department will determine detailed analyzing methods for the main efficacy indicators.

### Safety

As one of the modifications of the traditional acupuncture, TEAS is non-invasive and sends stimulation into acupoints through electrodes on the skin, therefore harm to the patient is limited. However, some adverse events such as treatment side effects, psychological harm or trauma, injury will be recorded. AEs should be recorded truthfully and in detail, containing clinical manifestation, occurrence time, severity, duration, related causes, related treatments, and outcomes. When AEs occur, the researchers must assess the event and discuss whether to withdraw the patients from the observation. All AEs should be treated timely and be followed until the individual recovering to a stable condition. Compensation will be provided to those who suffer harm from trial participation in accordance with hospital regulations.

## Discussion

To our best knowledge, this will be the first randomized clinical trial comparing TEAS with SA in the treatment of postoperative pain in patients undergoing Endoscopic submucosal dissection (ESD). We try to prove the hypothesis that compare with SA, TEAS may alleviate postoperative pain and enhance gastrointestinal function recovery in patients after ESD.

Postoperative pain after ESD is a very common and non-lethal complication, which endoscopists always take for granted. Some previous studies reported an incidence rate of 42.3%-98% of post-ESD pain [17, 18]. Jung et al. reported that the incidence of moderate to severe pain requiring pain medication after ESD was 53.8% [19]. Similarly, a retrospective study about ESD pain found that 36.4% patients needed a pain reliever after ESD [4]. Although post ESD-pain is usually transient and self-limiting, it is a key factor resulting in worse life quality and higher medical costs [17, 20, 21]. Additionally, post ESD-pain will lead to negative emotion of patients towards the treatments. Since ESD is an organ protective operation, metachronous lesions are an important issue in monitoring [22]. Repetitive ESD surgeries are very common for patients even if they underwent the radical resection [7]. Patients who have experienced postoperative pain after ESD may doubt the subsequent endoscopic surgeries or the treatments of recurrent lesions. Therefore, it is significant for patients undergoing ESD to have sufficient, effective prevention and management of postoperative pain, which will highly improve the patients’ satisfaction and compliance for further treatments and monitoring.

According to previous studies results, female gender, long operation time, lesion location and acid allergy are the potential risk factors of post ESD pain [4, 18, 19]. Although without fully understanding of the mechanism of the post ESD pain, some studies shows that it may be associated with residual mucosal defects (dropsical and inflamed artificial ulcers), transmural burns and air leaks [23, 24], acid sensitivity [19], or the penetration or chemistry of submucosal fluid injections [4]. Besides that, severe inflammatory reaction [25] and pulling of inflammatory tissue [19] may also be the mechanism of pain development, as well as bile acid reflux [26] and gas expansion [18] after ESD. Based on the factors and mechanisms above, we need to explore suitable postoperative analgesia for ESD surgery patients and to give preventive and early analgesic drugs for high-risk groups.

Currently, no unified conclusion is made on how to perform effective postoperative analgesia on patients with gastric and esophageal ESD surgery. Jung et al. suggested that hypersensitivity to gastric acid had large possibility to fuel the pain after ESD, and the prophylactic treatment of proton pump inhibitor (PPI) was effective in reducing pain [19]. Intravenous dexamethasone given after the ESD surgery was found helpful to alleviate epigastric postoperative pain [7]. Kim et al. proved that local infiltration treatment with bupivacaine and triamcinolone could also help relieve abdominal pain after ESD [8, 9]. Intravenous lidocaine during ESD can reduce the amount of intraoperative opioids and patients’ body movement, while relieving postoperative pain in the upper abdomen and throat [8].

Nowadays, many studies have demonstrated that perioperative acupuncture stimulation can reduce the amount of anesthetic drugs’ consumption [13], maintain hemodynamic stability, decrease postoperative pain, promote gastrointestinal function recovery [27, 28], and prevent nausea and vomiting [10]. One meta-analysis indicated that acupuncture or related techniques were associated with less postoperative pain and with significantly greater reduction in opioid analgesic consumption one day following the surgery compared with the control [12]. Another multicenter, randomized study demonstrated that electroacupuncture shortened the duration of postoperative ileus and promoted gastrointestinal function recovery [27]. Wang J, et al. also found that acupuncture could significantly reduce the incidence of adverse events, relieve patients’ pain, and improve patient satisfaction during colonoscopy [29]. However, the effect of acupuncture or TEAS on post ESD pain and gastrointestinal function recovery is still unknown.

In conclusion, this trail is expected to indicate that perioperative TEAS treatment may relieve postoperative pain and promote the recovery of gastrointestinal function for patients undergoing gastric and esophageal ESD procedures. This study aims at seeking a more suitable postoperative analgesia scheme for these patients and solving the current problems of imperfect postoperative analgesia managements and large adverse effects after ESD.

## Trial status

The first version was developed on October 10, 2021, and the first participant was recruited on December 1st, 2021; this is the first version, and the version No was V1.0/2021.10.10. The enrollment will be completed on December 30th, 2023. At present, 70 participants have been formally included, and the trial is still ongoing.

## Supplementary Information

**Additional file 1.** Trial registration Information.

**Additional file 2.** SPIRIT 2013 Checklist.

## Data Availability

After the study is completed and the results are published, the data and trial results will be open to the public (including the participates) through email connecting with the corresponding author.

## Abbreviations

ESD: Endoscopic submucosal dissection
VAS: Visual analog scale
NRS: Numeric Rating Scale
PONV: Postoperative nausea and vomiting
CRF: Case report forms
BMI: Body mass index
ASA: American Society of Anesthesiologists
CONSORT: Consolidated Standards of Reporting Trials
AEs: Adverse events
SPIRIT: Standard protocol items: Recommendations for interventional trials
PACU: Post-anesthesia care unit
DMC: Data Monitoring Committee
IRBs: Institutional Review Boards
NSAIDs: Non-steroidal anti-inflammatory drugs
