# Supplementary material for: Effects of transcutaneous electrical acupoint stimulation (TEAS) on postoperative pain in patients undergoing gastric and esophageal ESD surgery: a study protocol for a prospective randomized controlled trial
Source: BMC Complement Med Ther. 2023 Jul 20;23:253. doi: 10.1186/s12906-023-04075-9 (PMC10357617; doi:10.1186/s12906-023-04075-9)
Supplement: Supplementary file 1 — Additional file 1. Trial registration Information. [file 12906_2023_4075_MOESM1_ESM.docx]

| Additional Table 1. Trial registration Information | |
| --- | --- |
| Date category | Information |
| Primary registry and trial identifying number | [www.chictr.org.cn](http://www.chictr.org.cn) ChiCTR2100052837 |
| Date of registration in primary registry | November 6th, 2021 |
| Secondary identifying numbers | No |
| Source(s) of monetary or material support | Capital’s Funds for Health Improvement and Research, PR China (Grant No. CFH 2022-2-20210) |
| Primary sponsor | Capital’s Funds for Health Improvement and Research |
| Secondary sponsor(s) | Beijing Administration of Traditional Chinese Medicine |
| Contact for public queries | Li-Xin An, MD, Professor [Email: anlixin8120@163.com] |
| Contact for scientific queries | Li-Xin An, MD, Professor, Department of Anesthesiology, Beijing Friendship Hospital, Capital Medical University, Beijing, China. |
| Public title | Transcutaneous Electrical Acupoint Stimulation (TEAS) on postoperative pain in patients undergoing gastric and esophageal ESD surgery |
| Scientific title | Effects of Transcutaneous Electrical Acupoint Stimulation (TEAS) on postoperative pain in patients undergoing gastric and esophageal ESD surgery: a study protocol for a prospective randomized controlled trial |
| Countries of recruitment | China |
| Health condition(s) or problem(s) studied | Postoperative pain |
| Intervention(s) | Active comparator: TEAS stimulation on L14,PC6,ST36,ST37 |
|  | Placebo comparator: stimulation on four sham acupoints |
| Key inclusion and exclusion criteria | Ages eligible for study: 18-75years; Sexes eligible for study: both; Accepts healthy volunteers: no |
|  | Inclusion criteria:  1)Aged 18–75years, diagnosed at gastric or esophageal tumor undergoing ESD procedure, regardless of gender;  2)18 kg/m2≤BMI≤30 kg/m2;  3) ASA classification levels I–Ⅲ;  4)Participating in the study voluntarily and signing informed consent |
|  | Exclusion criteria:  1)Surgical incision or scar in the meridian of the acupoints of Hegu (L14), Neiguan (PC6), Shangjuxu (ST37), Zusanli (ST36).  2)Local skin infection at the acupoints above.  3)Upper limb or lower limb nerve damage.  4)Participating in other clinical studies within the past 4 weeks.  5)Inability to understand the numeric rating scale (NRS) score and the visual analog scale (VAS) score.  6)Application to a pacemaker.  7)Gravida, puerperant and patients with positive urine pregnancy tests.  8)Combined with preoperative pain, using central analgesic drugs, addicted to or dependent on opioids.  9)Preoperative complications including severe CNS diseases or severe mental diseases.  10)Considered not suitable for this study. |
| Study type | Interventional |
|  | Allocation: randomized; Intervention model: parallel assignment; Masking: single blind. |
|  | Primary purpose: prevention |
|  | Phase III |
| Date of first enrolment | March 2022 |
| Target sample size | 120 |
| Recruitment status | Recruiting |
| Primary outcome(s) | VAS score, the incidence of post-ESD VAS≥4, the consumption of morphine during follow-up |
| Key secondary outcomes | Anesthesia-associated parameters, sedation score, nausea and vomiting score, shivering score, the recovery of gastrointestinal function, satisfaction of patients, QLQ-C30 life quality scale, economic indicators |
